# A case of Mirizzi syndrome accompanied by a pseudoaneurysm that ruptured into the gallbladder: successfully treated by embolization of aneurysm and sequential surgery

**DOI:** 10.1186/s40792-022-01467-w

**Published:** 2022-06-14

**Authors:** Ryosuke Fukushima, Norihiro Ishii, Norifumi Harimoto, Kenichiro Araki, Akira Watanabe, Mariko Tsukagoshi, Kei Hagiwara, Takahiro Yamanaka, Ken Shirabe

**Affiliations:** 1 Department of General Surgical Science, Graduate School of Medicine, Gunma University, 3-39-22 Showa-machi, Maebashi, Gunma Japan; 2 Department of General Surgical Science, Division of Hepatobiliary and Pancreatic Surgery, Graduate School of Medicine, Gunma University, 3-39-22, Showa-machi, Maebashi, Gunma Japan

**Keywords:** Mirizzi syndrome, Ruptured pseudoaneurysm, Transcatheter arterial embolization, Cholecystectomy

## Abstract

**Background:**

Although visceral aneurysms are relatively rare, it can be life-threatening in case it ruptures. We report a case of Mirizzi syndrome accompanied by a pseudoaneurysm that ruptured into the gallbladder.

**Case presentation:**

The patient was a 73-year-old woman with persistent gastrointestinal bleeding and progressive jaundice. Examination revealed a pseudoaneurysm in the gallbladder artery or hepatic artery branch, and biliary hemorrhage due to gallbladder perforation was suspected. Urgent abdominal angiography revealed a pseudoaneurysm measuring 50 × 32 mm that had ruptured directly from the right hepatic artery or the cystic artery into the gallbladder. The pseudoaneurysm was successfully coiled and the bleeding was stopped. The presence of ongoing obstruction due to Mirizzi syndrome resulted in an emergency cholecystectomy being performed on the same day. On removing the impacted gallstone from the neck of the gallbladder, we found an obstruction between the lateral wall of the common bile duct and the gallbladder, this condition was diagnosed as Mirizzi syndrome with a biliobiliary fistula. After removing the impacted gallstone, a T-tube was inserted into the common bile duct. Bile leakage was observed postoperatively, but it improved with drainage. The patient fully recovered.

**Conclusions:**

We present our experience with a case of Mirizzi syndrome accompanied by a ruptured pseudoaneurysm successfully treated with coil embolization followed by cholecystectomy. In this case, the pseudoaneurysm may have been caused by inflammation due to cholecystitis or compression of the arterial wall by a gallstone. To the best of our knowledge, Mirizzi syndrome associated with pseudoaneurysm rupture is rare. Our study suggested that cholecystectomy preceded by transcatheter arterial embolization is an effective strategy to control bleeding in patients with hemobilia due to aneurysm.

## Background

The pathology of Mirizzi syndrome involves the presence of stones in the neck of the gallbladder or the cystic duct that compress the common hepatic duct, resulting in jaundice [1]. In contrast, splanchnic artery aneurysms are rare, with an incidence of 0.01–2% [2]; however, they can be life-threatening when they rupture. Here, we report a case of Mirizzi syndrome accompanied by a pseudoaneurysm that ruptured into the gallbladder, which was successfully treated through the embolization of the aneurysm and sequential surgery.

## Case presentation

A 73-year-old woman was hospitalized for cholecystitis due to gallstones, and computed tomography (CT) revealed dilatation of the intrahepatic bile ducts. Therefore, endoscopic retrograde cholangiopancreatography was performed for it. Moreover, substantial coagulation was found in the stomach. Although endoscopic hemostasis was performed for suspected gastrointestinal bleeding, the patient continued to have bloody stools, and the jaundice worsened. Enhanced abdominal CT showed extravasation in the gallbladder, and she was suspected to have hemobilia due to gallbladder perforation from the pseudoaneurysm found in the cystic artery or a branch of the hepatic artery. Subsequently, she was transferred to our hospital.

At the initial visit, she presented with right hypochondrium pain and jaundice of the skin and eyes. Blood tests revealed an increase in the levels of white blood cells, C-reactive proteins, and biliary enzymes. Her bilirubin levels were high at 14.3 mg/dl. Abdominal ultrasonography revealed a 50 × 30 mm cyst-like mass near the gallbladder, and a Doppler ultrasound indicated a beating signal. The gallbladder wall was also thickened and hyperintense, and the intrahepatic bile ducts in the left lobe of the liver were dilated.

**Fig. 1:**
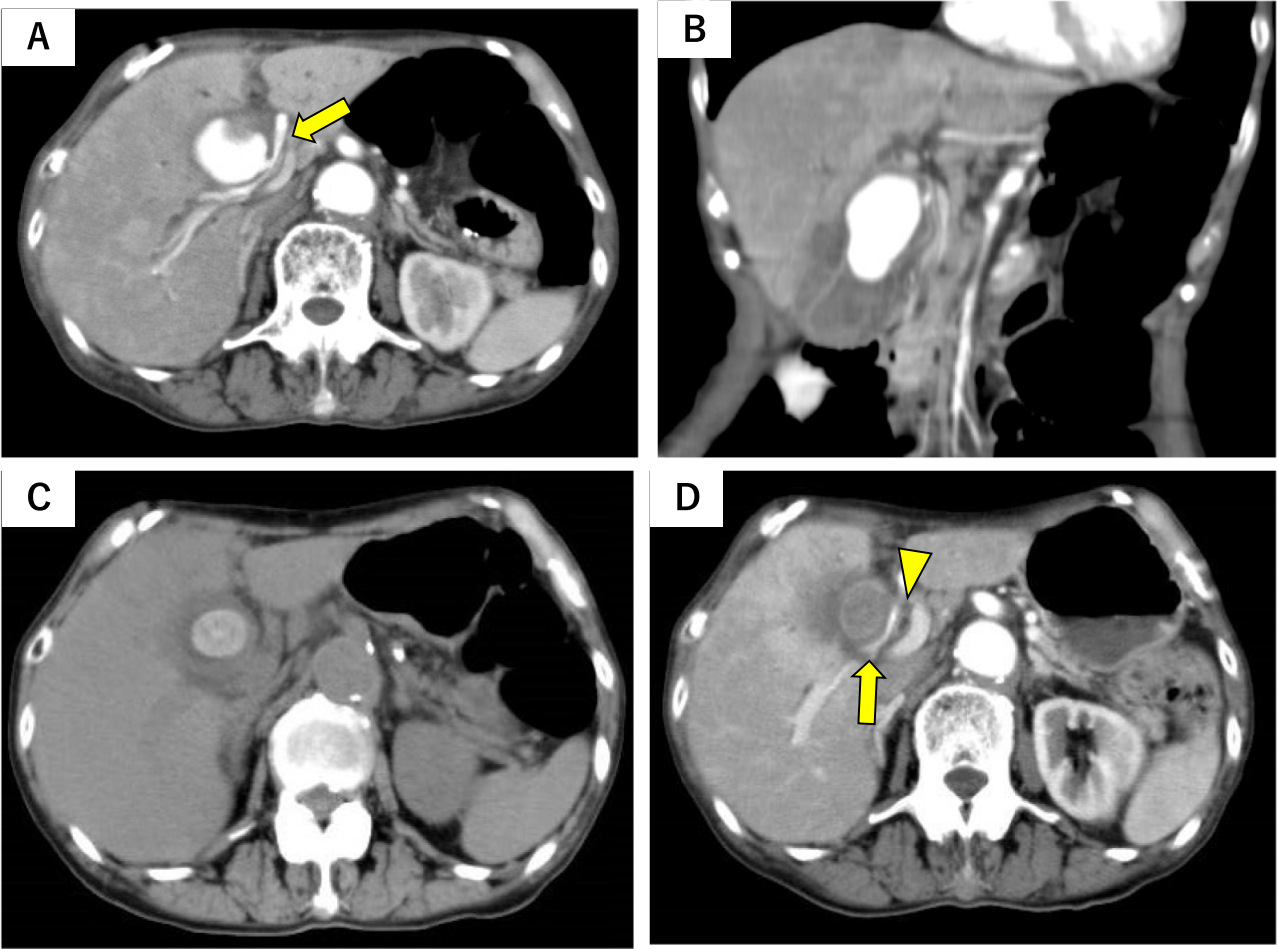
Enhanced abdominal computed tomography (CT) findings. Enhanced CT revealed a pseudoaneurysm in the gallbladder, which was suspected to be connected to the right hepatic artery or the cystic artery **A** (yellow arrow) axial view, **B** coronal view. Moreover, CT revealed a 20-mm gallstone in the neck of the gallbladder (**C**). The right hepatic artery (yellow arrow) runs ventral to the common bile duct (yellow arrowhead) and is in close proximity to the gallbladder (**D**)

Enhanced CT revealed a pseudoaneurysm in the gallbladder, which was suspected to be derived from the right hepatic artery or the cystic artery (Fig. 1A, B). Moreover, a 20-mm gallstone was observed in the neck of the gallbladder, and the gallbladder wall was edematous, indicating acalculous cholecystitis (Fig. 1C). In this case, the right hepatic artery ran ventral to the common bile duct and was in close proximity to the gallbladder (Fig. 1D).

Therefore, we diagnosed acute cholecystitis and hemobilia due to perforation of the gallbladder by a pseudoaneurysm. An urgent abdominal angiography was performed to identify the source of bleeding and control it.

**Fig. 2:**
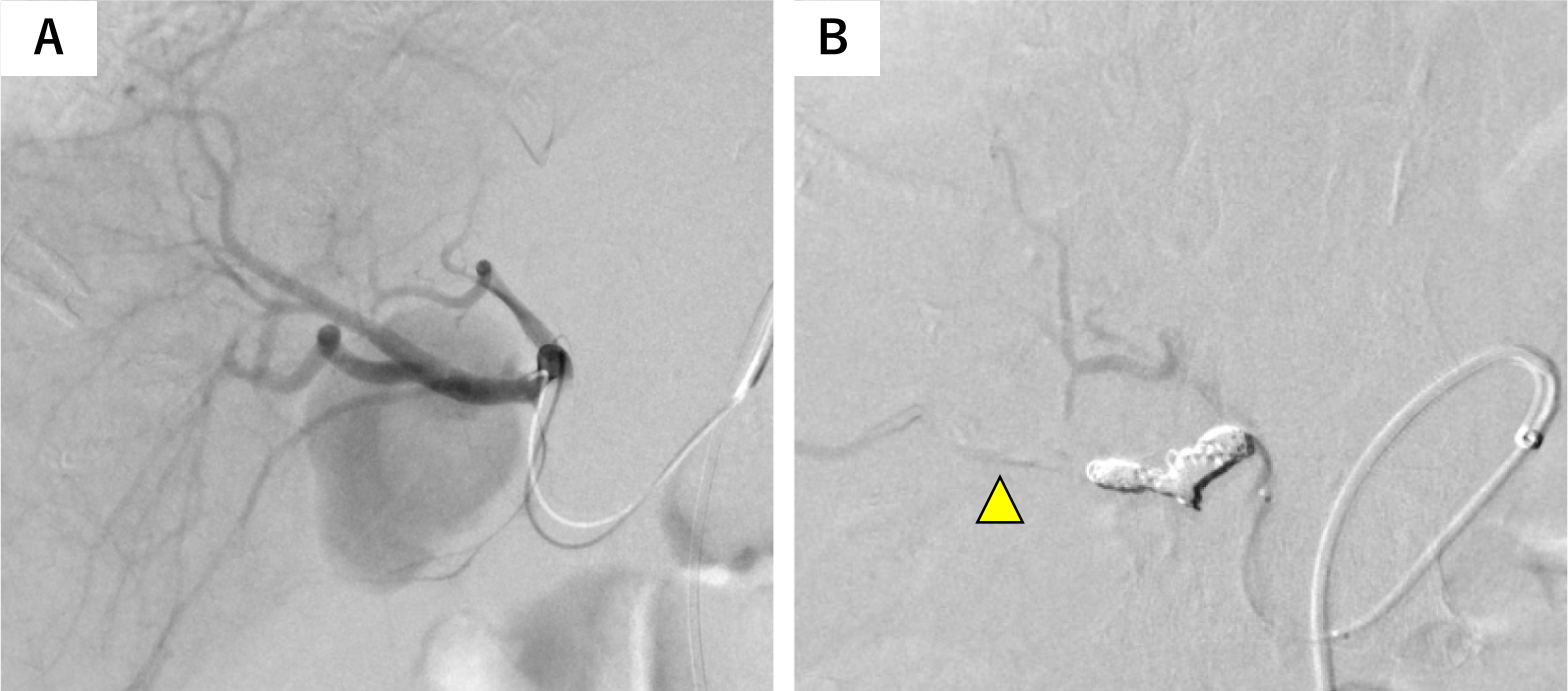
Angiography findings on admission and after transcatheter arterial embolization (TAE). Digital subtraction angiography from the right hepatic artery indicated a 50 × 32-mm pseudoaneurysm derived directly from the right hepatic artery or the cystic artery (**A**). We isolated the pseudoaneurysm by embolizing its proximal and distal portions of the pseudoaneurysm (**B**). The bleeding was stopped, and it was confirmed that the distal end of the right hepatic artery was supplied by the collateral blood flow of the middle hepatic artery (yellow arrowhead)

Digital subtraction angiography (DSA) from the right hepatic artery revealed a 50 × 32 mm pseudoaneurysm derived directly from the right hepatic artery or the cystic artery (Fig. 2A). We initially planned to place a stent to preserve right hepatic artery flow; however, stenting was difficult because of the meandering vessels and vasospasm. Moreover, we observed collateral artery flow from the middle hepatic artery; therefore, we prioritized coil embolization of the right hepatic artery to stop the bleeding. The pseudoaneurysm was isolated by embolizing its proximal and distal portions. DSA of the celiac artery after embolization indicated the absence of blood flow in the aneurysm and that the distal right hepatic artery was supplied by the collateral arterial flow of the middle hepatic artery (Fig. 2B).

We successfully stopped the bleeding with coil embolization, which was followed by an urgent open cholecystectomy for the possibility of rebleeding due to worsening inflammation caused by gallbladder infarction and persistent cholecystitis, as well as severe cholangitis due to hematoma in the bile duct.

### Intraoperative findings

**Fig. 3:**
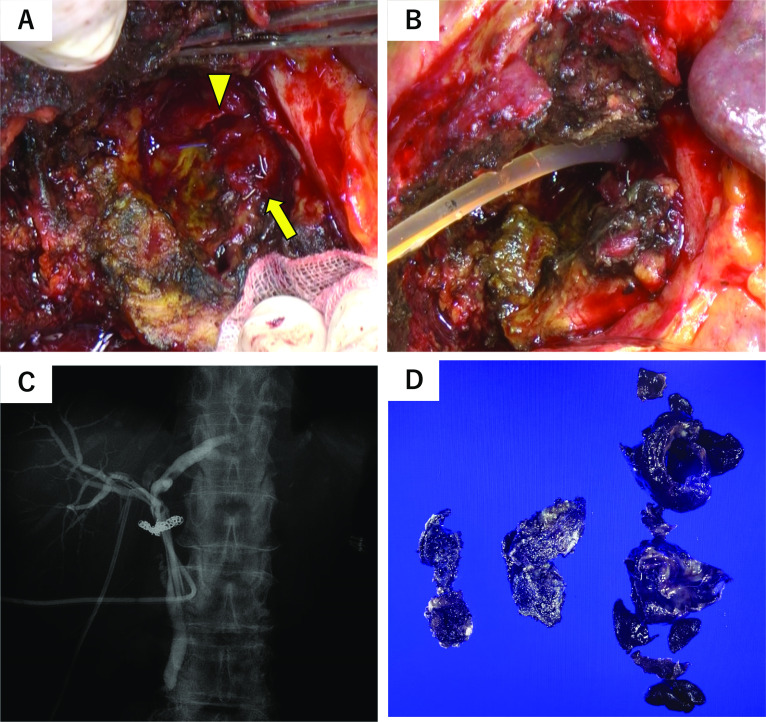
Intraoperative images, cholangiogram, and macroscopic findings of the resected gallbladder. Intraoperative image: the coil used for embolization of the right hepatic artery was observed in the neck of the gallbladder (**A**: yellow arrow). The biliobiliary fistula was also observed (**A**: yellow arrowhead). A T-tube was inserted into the common bile duct from the fistula (**B**). Cholangiogram: the peripheral bile ducts were visualized and no leakage from the bile ducts was observed (**C**). The exact size of the gallbladder could not be determined because it was resected as much as possible. The gallbladder wall was clearly thickened, with edema, fibrosis, and granulomatous changes. No malignant findings were observed (**D**)

Upon opening the abdomen, the gallbladder was found to be highly inflamed and distended. Hematomas were observed when the gallbladder wall was opened near the fundus. We proceeded to remove the abdominal side of the gallbladder and observed the coil used for embolizing the right hepatic artery in the neck of the gallbladder and bleeding from the same site (Fig. 3A). We sutured the bleeding point from within the gallbladder wall, which stopped the bleeding. In addition, when the gallstone impacted in the neck of the gallbladder was removed, the lateral wall of the common bile duct and the gallbladder were noted to be in contact, and the patient was subsequently diagnosed with Mirizzi syndrome with a biliobiliary fistula (Fig. 3A). A T-tube was inserted into the common bile duct from the fistula (Fig. 3B), and a cholangiogram was performed (Fig. 3C). The coil used for embolization was observed, so the bleeding was considered well controlled by transcatheter arterial embolization (TAE) with only partial deviation of coil. After the peripheral bile ducts were visualized and it was confirmed that there was no leakage, a drain was placed in the gallbladder bed, and the operation was completed.

### Histopathological findings

The gallbladder wall showed clear thickening, with edema, fibrosis, and granulomatous changes. Inflammatory granulation tissue was observed, comprising inflammatory cell infiltrates and fibroblasts. Most of the epithelium had fallen off, and the nuclei of the remaining epithelium were inflamed and enlarged. No malignant findings were observed (Fig. 3D).

### Postoperative course

Bile leakage from the site of insertion of the T-tube was observed after surgery, but improved with drainage. No hepatic infarction was observed after the embolization of the right hepatic artery. The drainage tube was removed on postoperative day 24, and the patient was discharged from the hospital on postoperative day 29. The T-tube was removed on postoperative day 60. The patient is currently under outpatient observation.

## Discussion

McSherry defined Mirizzi syndrome as a condition wherein the common hepatic duct may be stenotic because of mechanical compression or inflammatory changes caused by the impaction of stones in the neck of the gallbladder or the cystic duct [1]. The cystic duct and the common hepatic duct run parallel to each other, this anatomical feature supports the pathology, which is believed to occur more frequently when the cystic duct is long or when it joins the common bile duct at low positions [3]. Although differences exist in the classification systems advocated in different studies [3–5], Mirizzi syndrome can be mainly classified into two main types: type I, the classical type that involves pressure and stenosis and type II that involves a biliobiliary fistula due to necrosis of the bile duct resulting from compression caused by a stone in the gallbladder neck. Type II can be further subcategorized into type IIa wherein the stone forms a fistula with the bile duct at the neck of the gallbladder and type IIb wherein the stone impacts into the confluence of the common bile duct, and the cystic duct cannot be identified [1]. When selecting the surgical treatment, the size of the biliobiliary fistula must be considered: in types I and II with a relatively small fistula, it is common to resect the gallbladder as much as possible, remove the gallstones, and insert a T-tube. It has been reported that in cases of large or type IIb fistulas where the cystic ducts cannot be identified, choledochoplasty is performed by making an incision on the base of the gallbladder and covering the bile duct defect created by removal of the gallbladder using the wall of the gallbladder or a pedicled omentum [6]. Generally, the larger the biliobiliary fistula, the more difficult and complicated the biliary reconstruction and the more often it is necessary to perform bile duct resection or choledochojejunostomy through Roux-en-Y reconstruction [7, 8].

Based on the intraoperative findings in our case, it was considered as Mirizzi syndrome type II with a biliobiliary fistula. Accordingly, surgery was completed by inserting a T-tube after resecting the gallbladder as much as possible, leaving only a portion in the gallbladder bed.

In contrast, the incidence of abdominal visceral artery aneurysms is reported to be relatively rare at 0.01% − 2% [2]. Aneurysms are classified as either true aneurysms or pseudoaneurysms based on their form of occurrence. Pseudoaneurysms rupture more frequently than true aneurysms. Hepatic arterial pseudoaneurysms account for approximately 20% of all abdominal visceral artery aneurysms and are reported to rupture more frequently than others [2]. The intrahepatic hepatic artery accounts for 20% of all aneurysms, the extrahepatic hepatic artery accounts for 80%, and the common hepatic arteries account for 33%, followed by the right hepatic artery, the proper hepatic artery, and the left hepatic artery [9]. Most ruptures occur in the abdominal cavity or bile ducts. Occasionally, the aneurysms can rupture into the gastrointestinal tract or portal vein system, but ruptures into the gallbladder, as in this case, are rare. The right hepatic artery usually crosses the anterior portal vein, runs dorsal to the common bile duct, and then branches into anterior and posterior branches. In our case, the right hepatic artery ran ventral to the common bile duct and was in close proximity to the gallbladder, this anatomical feature may be the cause of the relatively rare perforation into the gallbladder (Fig. 1D).

The etiology of pseudoaneurysms includes trauma, iatrogenic like abdominal surgery and catheterization, inflammation, infection, tumor, and vascular anomalies. According to a recent review, the iatrogenic causes are increasing [9]. On the contrary, pseudoaneurysms caused by cholecystitis or Mirizzi syndrome have been rarely reported.

Considering the clinical course and intraoperative findings, the cause of the pseudoaneurysm in our patient was believed to be chronic inflammation caused by cholecystitis and arterial injury caused by mechanical compression by the stone. The patient had a history of cholecystitis, which had occurred 4 months earlier, and was treated conservatively. The intraoperative findings revealed that the gallbladder was highly inflamed and adherent to the surrounding organs, such as the transverse colon and duodenum, suggesting widespread severe inflammation caused by cholecystitis. In addition, a gallstone in the neck of the gallbladder was found to form a biliobiliary fistula with the common bile duct, and the stone was expected to compress the artery. In this case, the right hepatic artery or the cystic artery, which was weakened by cholecystitis-related inflammation, was mechanically stimulated by the gallstone, which resulted in the formation of a pseudoaneurysm. In case of metallic bile duct stenting for endoscopic biliary drainage, a pseudoaneurysm in the right hepatic artery is more common because it is easily irritated by the metal wire at the stent end [10].

The curative treatment for cholecystitis and Mirizzi syndrome complicated by hemobilia and hepatic aneurysm, such as in this case, is basically cholecystectomy because of the possibility of recurrent hemorrhage and carcinoma development [11–14]. It has recently been reported that TAE is minimally invasive, can be performed following angiographic diagnosis, and that control of bleeding by TAE is accomplished prior to radical surgery [15–17].

Although no consensus has been established regarding the timing of radical surgery after TAE, many reports indicate that the timing of treatment for acute cholecystitis should be determined according to the Guidelines for the Treatment of Acute Cholangitis and Cholecystitis 2018 [18]. At the very least, it is reasonable to perform radical surgery before inflammation resolves and before the adhesions between the gallbladder and the surrounding tissue become strong. Based on the many case reports of Mirizzi syndrome with biliobiliary fistulas, elective surgery is common. The surgery timing should be determined by detailed preoperative imaging and pathological diagnosis, including cytodiagnosis, and by considering planned surgery for both benign diseases, such as cholelithiasis, and malignant diseases, such as gallbladder cancer and hilar cholangiocarcinoma, or their associated complications [19]. Although it is often difficult to preoperatively evaluate whether a biliobiliary fistula is present, in cases of bile duct stenosis or jaundice, endoscopic retrograde cholangiography and endoscopic nasobiliary drainage tube placement before surgery are reportedly effective [20]. Regardless of the presence or absence of a biliobiliary fistula, it is necessary to consider the possibility of malignant disease and highly invasive surgery in Mirizzi syndrome and to decide on the surgery timing after thorough preoperative discussion and stabilization of the patient's condition.

In the present case, we considered the possibility of worsening of inflammation and necrosis of the gallbladder due to gallbladder infarction, rebleeding due to persistent inflammation, and severe cholangitis due to hematoma in the bile duct; consequently, we performed emergency surgery on the same day. Although surgery is the first choice for Mirizzi syndrome, TAE has been reported to be effective for aneurysms associated with hemobilia [15–17]. And in this case, we performed cholecystectomy after TAE. We believe that a strategy of cholecystectomy preceded by TAE may be a safe and effective strategy to control bleeding, in patients with hemobilia due to aneurysm. The operative findings revealed that the patient had Mirizzi syndrome with a biliobiliary fistula and no malignancy, and thus, surgery was completed with cholecystectomy plus T-tube placement only.

When we perform TAE, we must consider organ ischemia after embolization. The liver receives dual blood flow controlled by the hepatic artery and portal vein, and embolization of the hepatic artery can be performed safely if portal vein blood flow is maintained. Generally, it has been reported that hepatic infarction rarely occurs as the liver contains well-developed collateral blood channels [21]. However, hepatic infarctions can be fatal [22], and the location of the aneurysm and the anatomy of the arterial system must be fully considered before deciding the treatment strategy. At the common hepatic artery level, blood flow to the liver is maintained via the right gastric artery, gastroduodenal artery, and superior mesenteric artery arcade, and the risk of hepatic ischemia is considered low [23]. It has been reported that TAE at the level of the common hepatic artery can be safely performed without postoperative hepatic infarction or hepatic failure [24]. Similarly, as the right hepatic artery and left hepatic artery are connected via the hepatic artery traffic branch, even if one of them is embolized, blood flow to the liver is retained and the risk of inducing postoperative hepatic ischemia is also low [25]. In constant, for proper hepatic artery aneurysms and common hepatic artery aneurysms extending to the gastroduodenal artery, reconstruction as well as aneurysm resection is required to avoid liver failure due to decreased hepatic blood flow [26]. In our case, the right hepatic artery was embolized with the coiling, but DSA from the celiac artery confirmed the peripheral right hepatic artery by collateral arterial flows from the middle hepatic artery, which suggests that hepatic artery blood flow was secured.

## Conclusions

We present our experience with a case of ruptured pseudoaneurysm accompanied by Mirizzi syndrome. We first performed angiography to confirm the localization of the pseudoaneurysm and control bleeding, followed by coil embolization of the right hepatic artery. We successfully stopped the bleeding through coil embolization, after which urgent open cholecystectomy was performed because of the possibility of rebleeding due to worsening inflammation caused by gallbladder infarction and persistent cholecystitis as well as severe cholangitis due to hematoma in the bile duct.

Postoperatively, the patient was discharged without hepatic infarction after embolization of the right hepatic artery. To the best of our knowledge, Mirizzi syndrome associated with pseudoaneurysm rupture is rare. An effective strategy is to precede TAE with cholecystectomy in patients who can be treated safely without postoperative complications.

## Data Availability

Data sharing is not applicable to this article as no datasets were generated or analyzed during the current study.

## Abbreviations

CT: Computed tomography
ERCP: Endoscopic retrograde cholangiopancreatography
DSA: Digital subtraction angiography
TAE: Transcatheter arterial embolization

## References

[1] McSherry CK, Ferstenberg H, Virship M (1982). The Mirizzi syndrome : suggested classification and surgical therapy. Surg Gastroenterol.

[2] Stanley JC, Wakefield TW, Graham LM, Whitehouse WM, Zelenock GB, Lindenauer SM (1986). Clinical importance and management of splanchnic artery aneurysms. J Vasc Surg.

[3] Clemett AR, Lowman RM (1965). The roentgen features of the Mirizzi syndrome. Am J Roentgenol.

[4] Corlette MB, Bismuth H (1975). Biliobiliary fistula. A trap in the surgery of cholelithiasis. Arch Surg.

[5] Csendes A, Diaz JC, Burdiles P, Maluenda F, Nava O (1989). Mirizzi syndrome and cholecystobiliary fistula: a unifying classification. Br J Surg.

[6] Ebata T, Takagi K, Nagino M (2011). Hilar cholangioplasty using omentum for ductal defect in biliobiliary fistula. J Hepato Bil Pancreat Sci.

[7] Igarashi R, Irisawa A, Shibukawa G (2017). A case of Mirizzi syndrome with biliobiliary fistula (Corlette type I, Csendes type III). JJBA..

[8] Ishizuka J, Umakoshi M, Kobayashi Y (2015). A case of Mirizzi syndrome with a biliobiliary fistula. J Jpn Surg Assoc.

[9] Uno K, Nakajima M, Yasuda K, Cho E, Mukai H, Hayakumo T (1994). A case of hepatic artery aneurysm ruptured into the biliary tract to be treated with hepatic artery embolization. J Jpn Soc Gastroenterol.

[10] Yamaura M, Fukuda K, Mori K, Hirose S, Sato M, Endo M (2019). Two cases of hemobilia caused by a rupture of pseudoaneurysm. JJBA.

[11] Maeda A, Kunou T, Saeki S, Aono K, Murata T, Niionmi N (2002). Pseudoaneurysm of the cystic artery with hemobilia treated by arterial embolization and elective cholecystectomy. J Hepato-Bilia-Pancreat Surg.

[12] Kwan B, Waters PS, Olive E, Nathanson A, Bain R (2021). Haemobilia due to a ruptured right hepatic pseudoaneurysm secondary to Mirizzi syndrome with simultaneous cholecystoduodenal fistula. ANZ J Surg.

[13] Shishido Y, Fujimoto K, Yano Y, Mitsuoka E, Komatsubara T, Shio S, Ishii M, Higashiyama H (2021). Emergency surgery for hemobilia due to hepatic artery pseudoaneurysm rupture complicated by Mirizzi syndrome type II: a case report. BMC Surg.

[14] Lin SZ, Tseng CW, Chen CC (2009). Hepatic artery pseudoaneurysm presenting with Mirizzi syndrome and hemobilia. Clin Gastroenterol Hepatol.

[15] Srivastava DN, Sharma S, Pal S (2006). Transcatheter arterial embolization in the management of hemobilia. Abdom Imaging.

[16] Anderson O, Faroug R, Davidson BR, Goode JA (2008). Mirizzi syndrome associated with hepatic artery pseudoaneurysm: a case report. J Med Case Rep.

[17] Suzuki S, Saito Y, Nakamura K, Sukegawa R, Chiba A, Nakajima S (2013). Unruptured cystic artery pseudoaneurysm accompanied by Mirizzi syndrome: a report of a case. Clin J Gastroenterol.

[18] Takada T (2018). Tokyo Guidelines 2018:updated Tokyo guidelines for the management of acute cholangitis / acute cholecystitis. J Hepatobiliary Pancreat Sci.

[19] Tokunaga Y, Sasaki H, Matsusige S (2009). A case of Mirizzi syndrome with a biliobiliary fistula (Corlette type I, Csendes type II). J Biliary Tract Pancreas.

[20] Miura K, Sodeyama H, Nakata S (2012). A surgical case of cholecystolithiasis with fistula formation at the gallbladder and the posterior segmental branch. J Jpn Soc Clin Surg.

[21] Plengvanit U, Chearanai O, Sindhvananda K, Dambrongsak D, Tuchida S, Viranuvatti V (1972). Collateral arterial blood supply of the liver after hepatic artery ligation, angiographic study of twenty patients. Ann Surg.

[22] Okuno A, Miyazaki M, Ito H, Ambiru S, Yoshidome H, Shimizu H (2001). Nonsurgical management of ruptured pseudoaneurysm in patients with hebatobiliary pancreatic diseases. Am J Gastroenterol.

[23] O’Driscoll D, Olliff SP, Olliff JF (1999). Hepatic artery aneurysm. Br J Radiol.

[24] Baggio E, Migliara B, Lipari G, Landoni L (2004). Treatment of six hepatic artery aneurysms. Ann Vasc Surg.

[25] Gunji H, Cho A, Tohma T (2006). The blood supply of the hilar bile duct and its relationship to the communicating arcade located between the right and left hepatic arteries. Am J Surg.

[26] Eto E, Ito Y, Mihara K, Egawa T, Hayashi S, Nanashima A (2013). Three case reports of hepatic artery aneurysm treated by surgical and endovascular treatment. J Jpn Soc Clin Surg.

